# Flux-tunable phase shifter for microwaves

**DOI:** 10.1038/s41598-017-15190-2

**Published:** 2017-11-07

**Authors:** Roope Kokkoniemi, Tuomas Ollikainen, Russell E. Lake, Sakari Saarenpää, Kuan Y. Tan, Janne I. Kokkala, Ceren B. Dağ, Joonas Govenius, Mikko Möttönen

**Affiliations:** 1 QCD Labs, COMP Centre of Excellence, Department of Applied Physics, Aalto University, FI-00076 Aalto, Finland; 2 National Institute of Standards and Technology, Boulder, Colorado 80305 USA; 3 Physics Department, University of Michigan, 450 Church St., Ann Arbor, MI 48109-1040 USA

## Abstract

We introduce a magnetic-flux-tunable phase shifter for propagating microwave photons, based on three equidistant superconducting quantum interference devices (SQUIDs) on a transmission line. We experimentally implement the phase shifter and demonstrate that it produces a broad range of phase shifts and full transmission within the experimental uncertainty. Together with previously demonstrated beam splitters, this phase shifter can be utilized to implement arbitrary single-qubit gates for qubits based on propagating microwave photons. These results complement previous demonstrations of on-demand single-photon sources and detectors, and hence assist in the pursuit of an all-microwave quantum computer based on propagating photons.

## Introduction

Since its initial theoretical considerations in the 1980s^1^, quantum computing has been an active area of research thanks to its envisioned superior performance in certain computational problems^2–4^. However, the realization of a high-fidelity large-scale qubit register^5^ remains a major challenge. Among many proposals^6^, propagating photons constitute an interesting candidate for a quantum register since they exhibit weak decoherence^7^ and photons can be directly used for fast and secure communication^8^.

In addition to a universal set of quantum gates^9^, a photonic quantum computer calls for high-fidelity single-photon sources and detectors for qubit initialization and readout. In the optical regime, on-demand single-photon sources have reached 65% efficiencies^10,11^ (see also refs^12^ and^13^) and single-photon detectors are commercially available. Traditionally, tunable optical phase shifters have been slow to operate^14–16^, but recent electro-optical phase shifters^17^ have potential for 100-GHz operation^18^. However, large bias voltages or relatively long electrodes are needed for reasonable phase shifts^19,20^, hindering the scalability of the optical quantum computer. Furthermore, optical photons interact relatively weakly with nonlinear matter, making it very challenging to implement deterministic two-qubit gates. Measurement-based gates can provide an effective nonlinearity, but at the expense of adding severe overheads^21–24^.

Recent years have witnessed great progress in the implementation of high-fidelity superconducting qubits operating at microwave frequencies^5,25–32^. These nonlinear circuit elements can be engineered to interact very strongly^33^ with single photons^34–36^, providing opportunities for introducing deterministic photon–photon interactions^37^. In practice, on-demand single-photon sources in the microwave regime have already reached above 80% efficiencies^38–40^, thus surpassing the optical sources. Qubit-based single-photon microwave detectors^41–50^ have recently reached a quantum efficiency of 66%^51^ and calorimetric detectors^52,53^ have taken a leap towards the single-photon regime^54^. Owing to fully compatible fabrication techniques, all these components can be conveniently integrated on the same chip^35^, thus rendering propagating microwave photons an attractive alternative to optical wavelengths in realizing photonic quantum computing and communications.

However, no quickly tunable, compact, and high-fidelity phase shifter for propagating microwave photons has been demonstrated to date. A transmission line with a current-tunable kinetic inductance can be used as a phase shifter^55,56^, but it introduces significant reflections owing to mismatched characteristic impedances. Very recently, a tunable on-chip phase shifter was demonstrated^57^ with greatly improved impedance matching, but it still exhibits several decibels of loss and requires a microwave pump tone.

In addition to the implementation of photonic quantum gates, tunable phase shifters are useful in the on-chip integration of the control and measurement devices of a superconducting quantum computer. They may, for example, be used to tune the phase of on-chip coherent microwave sources^58^, thus possibly providing access to a universal set of single-qubit gates with a single off-chip source.

In this Report, we demonstrate a compact phase shifter based on three equidistant superconducting quantum interference devices (SQUIDs) interrupting a transmission line. We show that if the fluxes are chosen properly, the system exhibits full transmission with flux-tunable phase shift. At 6.3-GHz signal frequency, we experimentally obtain a phase shift range of size 3*π*/8, which is expected to increase with optimization of the sample parameters. Hence, our phase shifter conveniently expands the toolbox of superconducting quantum technology. In principle, the SQUIDs can be tuned *in situ* at timescales set by their plasma frequency which is typically higher than 10 GHz. Importantly, the phase shifter does not require a pump tone to operate.

## Results

**Figure 1:**
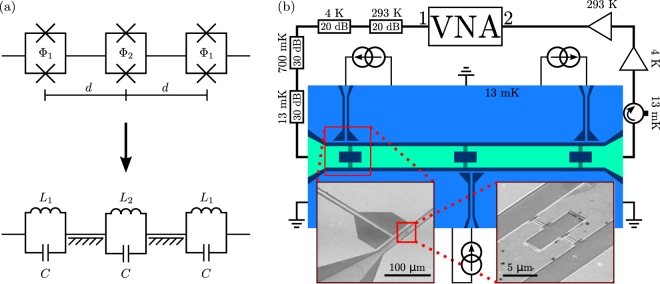
(**a**) Schematic diagram of the phase shifter composed of three SQUIDs separated by transmission lines of length *d* (top) and the corresponding electric-circuit model (bottom). Here, the SQUID magnetic flux is denoted by Φ_*k*_, Josephson inductance by *L*_*k*_, and capacitance by *C*. (**b**) Schematic illustration of the experimental sample and the measurement setup. The measurement signal is guided from the Port 1 of the vector network analyzer (VNA) to the sample through attenuators at four different temperatures as indicated. The output signal from the sample is amplified with two amplifiers, one inside the cryostat and one outside, before arriving at the VNA Port 2. The sample is protected from amplifier noise with an isolator. The three current sources allow us to control the flux bias of each SQUID individually. Here, niobium is denoted with light blue, aluminum with light green, Josephson junctions with dark green, and the substrate with dark blue color. The features are not to scale. We also show scanning electron microscope images of the flux bias scheme (left inset) and of the first SQUID (right inset).

First, we present an analytical expression for the transmission coefficient of the SQUID-based phase shifter. These results may be obtained using both classical circuit analysis and the quantum network theory^59^ which we employ in our derivation given in the Supplemental Material. We model the three SQUIDs forming the phase shifter as linear *LC* oscillators (see Fig. 1(a)), which is justified by the fact that the electric current induced by the microwaves is well below the typical critical currents of the SQUIDs which are tuned sufficiently far away from half integer flux values. The input current to the sample is of the order of a nanoampere, whereas the zero-flux critical current of the junctions is of the order of a microampere. Furthermore, we estimate from circuit simulations that the external quality factor of the SQUID chain is less than 10, and thus the linearity is well justified^60^.

The Josephson inductances of the SQUIDs are given by

1 $${L}_{i}=\frac{{{\rm{\Phi }}}_{0}}{4\pi {I}_{{\rm{c}},i}|\cos \,(\pi {{\rm{\Phi }}}_{i}/{{\rm{\Phi }}}_{0})|},$$

where Φ_0_ is the magnetic flux quantum, *I*_c,*i*_ and Φ_*i*_ are the zero-flux single-junction critical current and the magnetic flux threading the *i*th SQUID. There is also parallel capacitance, *C*, in the model arising from the junction capacitance and possible stray capacitances between the different sides of the SQUID. For simplicity, we have assumed that all Josephson junctions are identical and that the loop inductances of the SQUIDs are negligible.

We tune the inductances of the SQUIDs at the left and right ends of the chain to be equal, *L*_1_. The middle SQUID inductance is denoted by *L*_2_. Dissipation is negligible in the short superconducting waveguides we consider since quality factors over a million have been demonstrated with aluminum resonators^61^. Hence, we do not account for dissipation in our model. After a straightforward calculation, we find that the forward transmission coefficient for the voltage through the SQUID chain, *S*_21_(*ω*), is given by

2 $${S}_{21}(\omega )=\tfrac{[8{Z}_{0}^{3}{e}^{-2i\phi }\,(C{L}_{2}{\omega }^{2}-1)\,{(C{L}_{1}{\omega }^{2}-1)}^{2}]\,{[2{Z}_{0}(C{L}_{1}{\omega }^{2}-1)+i{L}_{1}\omega ({e}^{-2i\phi }-1)]}^{-1}}{4{Z}_{0}^{2}\,(C{L}_{2}{\omega }^{2}-1)\,(C{L}_{1}{\omega }^{2}-1)+{L}_{2}{L}_{1}{\omega }^{2}\,({e}^{-2i\phi }-1)-2i\omega {Z}_{0}\,[C{L}_{2}{L}_{1}{\omega }^{2}\,\mathrm{(2}+{e}^{-2i\phi })-{L}_{2}-{L}_{1}(1+{e}^{-2i\phi })]}.$$

Here, *Z*_0_ is the characteristic impedance of the transmission line, *ω* is the angular frequency of the incoming photons, *φ* = *ωd*/*v* is the phase shift due to a uniform transmission line of length *d*, and *v* is the speed of the wave in the transmission line.

If we define a free real-valued parameter *θ*, and restrict our attention to combinations of *L*_1_ and *L*_2_ that satisfy the ansatz

3 $${L}_{1}=\frac{2{Z}_{0}\,\sin (\theta /2)}{\omega \mathrm{[2}C\omega {Z}_{0}\,\sin \,(\theta /2)+\,\cos \,(\theta /2-2\phi )-\,\cos \,(\theta /2)]},$$

and

4 $${L}_{2}=\frac{4{Z}_{0}\,\sin \,(\theta /2)\,\cos \,(\theta /2-2\varphi )}{\omega \,\{2C\omega {Z}_{0}\,[\,\sin \,(\theta -2\phi )+\,\sin \,(2\phi )]-\,\cos \,(2\phi )+1\}},$$

we find that equation (2) reduces into

5 $${S}_{21}(\omega )={e}^{i(\theta -2\phi )}.$$

Thus choosing *L*_1_ and *L*_2_ according to this ansatz leads to full transmission (|*S*_21_| = 1) and the parameter *θ* to be the excess phase shift due to the SQUIDs. In practice, the achievable range of phase shifts is determined by the available values for the inductances and the employed photon frequency. For some combinations of *ω* and *θ*, equations (3) and (4) require negative inductances, and thus full transmission cannot be achieved. However, this limitation may be overcome using more than three SQUIDs in the phase shifter. A connection of *N* phase shifters in series trivially increases the range of tunability by a factor of *N*. In practice, some of the SQUIDs from such a long chain can be dropped, but we leave this optimization for future work.

Schematic illustration of our experimental sample realizing the three-SQUID phase shifter is shown in Fig. 1(b). The bonding pads for the center conductor of the waveguide are located near the left and right edges of the chip. Three broadband superconducting transmission lines provide tunable flux biases for each SQUID. The sample is cooled down in a commercial cryostat with a base temperature of 13 mK and measured according to the scheme presented in Fig. 1(b). We measure the transmission coefficient with a vector network analyzer operating in the low-power regime where the response is linear.

We normalize the measured transmission coefficient by a reference signal, for which all three SQUIDs are biased at an integer multiple of a flux quantum. This reference point is chosen since the SQUID inductances are minimized here, and hence they have a minimal effect on the transmitted signal. Namely, the zero-flux critical current of each SQUID is of the order 1 *μ*A, resulting in an impedance *iωL*_*k*_ ≈ *i* × 7Ω at the frequencies of interest. Hence, the estimated transmission amplitude for the circuit at the reference point is sufficiently close to unity, |*S*_21_| ≈ 0.98.

**Figure 2:**
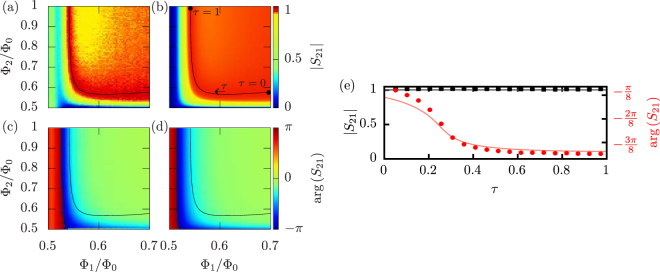
(**a**–**d**) Normalized scattering parameter of the SQUID-based linear phase shifter, *S*_21_, as a function of the flux through the side SQUIDs, Φ_1_, and through the middle SQUID, Φ_2_, at 6.3-GHz signal frequency. We show the measured (**a**) magnitude and (**c**) phase of the scattering parameter together with the corresponding theoretical predictions in panels (**b**) and (**d**), respectively. We define *τ* as the relative length along the curve of theoretical full transmission (solid line) which vanishes at (Φ_1_ = 0.7, Φ_2_ ≈ 0.58) and is unity at (Φ_1_ ≈ 0.54, Φ_2_ = 1) as indicated in panel (**b**). Note the non-standard colormaps in panels (**a–d**). (**e**) The amplitude (black color) and phase (red color) of the measured (markers) and theoretically obtained (solid lines) scattering parameters along the full-transmission curve. The measured values are obtained by fitting a quasilinear plane to the points at the curve in (**c**) and their eight nearest neighbors. The error bars denote the root-mean-square deviations from the fit. For the theoretical calculations, we set the zero-flux critical current of the middle SQUID to *I*_c,2_ = 2.2 *μ*A, that of the left and right SQUIDs to *I*_c,1_ = 0.7 *μ*A, and the SQUID capacitance to *C* = 26 fF.

The normalized magnitude and the phase of the measured transmission coefficient at 6.3-GHz frequency together with the corresponding theoretical predictions from equation (2) are shown in Fig. 2(a–d). We observe nearly full transmission over a wide range of flux values. Full transmission is obtained in the theoretical calculations along the solid line in Fig. 2(b). Thus we show in Fig. 2(e) the normalized magnitude and the phase of the transmission coefficient along this line. We clearly achieve a tunable phase shift with essentially unit transmission. The small discrepancy between the observed and the theoretical phase shifts is attributed to slight deviations from the assumption in the theory that the zero-flux critical currents of the side SQUIDs are equal. We note that the critical current of the side SQUIDs is roughly 90 nA for flux Φ_1_/Φ_0_ = 0.54, and hence the approximation of replacing the SQUIDs by linear *LC* oscillators holds along the full transmission curve.

**Figure 3:**
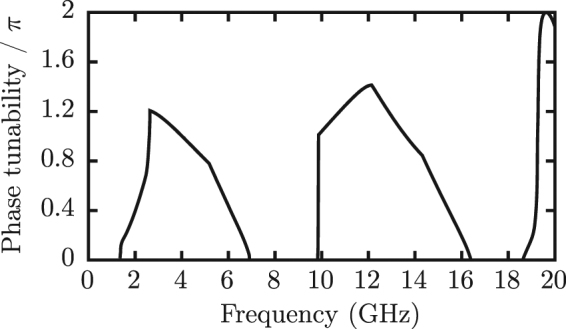
Theoretically predicted difference between the maximum and minimum phase shifts achieved for inductances *L*_1_ and *L*_2_ which are larger that their minimum values Φ_0_/(4*πI*_c,*i*_) but less than 10 nH as a function of signal frequency. The parameters are chosen as in Fig. 2.

To obtain insight on the operational characteristics of the phase shifter at different signal frequencies, we numerically solve from equations (3) and (4) the values of *θ* for which the inductances are in the experimentally achievable range for the realized sample. The results shown in Fig. 3 indicate that broad ranges of phase shifts are achievable in wide frequency bands. However, there are frequencies, for which all feasible inductance values lead to some reflections. Fortunately, these forbidden frequencies depend on the distance between the SQUIDs, and hence can be chosen in the fabrication.

## Discussion

In summary, we demonstrated theoretically and experimentally that a transmission line interrupted by three SQUIDs exhibits full microwave transmission with a tunable phase shift. We measured a tunability of 3*π*/8 at 6.3-GHz signal frequency and showed that this is in good agreement with the theory. Note that, in principle, the phase shifter works also if it is implemented with bulk components, e.g., variable inductors and coaxial cables, and operated at room temperature. However, there are many different phase shifter realizations available for room temperature applications^62–66^, and hence we choose to focus on the on-chip superconducting phase shifter owing to its direct applicability in quantum computing and technology. Our phase shifter thus opens horizons for future realizations of arbitrary single-qubit gates for propagating microwave photons, if integrated with nontunable beam splitters^35^. Another interesting future direction is the extension of this work to non-linear phase shifters and entangling two-qubit gates, which together with the single-qubit gates, can possibly be used to implement arbitrary many-qubit gates^67^.

The realization of a many-qubit quantum processor based on propagating microwave photons remains a challenge in the field of quantum technology. In the short term, implementation of arbitrary tunable single-qubit gates and integrated circuits including sources and detectors on the same chip seems plausible. Extension of the three-SQUID design, used here as a tunable phase shifter, to five or seven SQUIDs may allow an even broader range of phase shifts and signal frequencies. As a first step towards this goal, we aim to measure the three-SQUID phase shifter at multiple frequencies to demonstrate a much broader range of achievable phase shifts.

## Methods

### Sample fabrication

The sample is fabricated on an oxidized silicon substrate using standard lithographic techniques. The niobium ground plane and the flux bias lines are fabricated with optical lithography and the center conductor, along with the SQUIDs, are fabricated using electron beam lithography. The Al/AlO_x_/Al tunnel junctions are evaporated simultaneously with the center conductor to guarantee galvanic connection.

### Inductance matrix

We bias the SQUIDs with direct-current (dc) sources. Due to vanishing impedance of superconductors at dc, the bias current does not necessarily flow to the ground via the seemingly shortest available path. We observe this as significant cross coupling between the flux bias lines, i.e., applying current to one flux bias line changes the flux threading through all three SQUIDs. However, we must be able to address individual SQUIDs in the experiments. Hence, we quantify the cross coupling by measuring the inductance matrix.

To this end, we attach a superconducting solenoid to the middle of the outer surface of the sample holder, but inside the magnetic shield. The solenoid is used to provide a nonuniform flux bias to all three SQUIDs. If we measure the transmission through the phase shifter as a function of the current through the solenoid, we observe dips in the transmission at certain currents. Due to the nonuniformity, the dips occur at three distinct periods. Since the solenoid is attached to the middle of the sample holder, and thus to the middle of the sample, we expect the SQUID at the middle of the chain to have the shortest period. The periods of the remaining two SQUIDs can be conveniently determined after having measured the full inductance matrix between the on-chip SQUID coils and loops.

Applying current through one of the flux bias lines causes the dips in transmission shift to new solenoid current values. Measuring this shift for each SQUID and flux bias line allows us to determine the elements of the inductance matrix. We find the elements to be

6 $$M=(\begin{array}{ccc}-0.93 & 1.47 & -6.87\\ -4.19 & -2.27 & -4.67\\ -0.30 & -0.06 & -6.13\end{array})\times {10}^{2}\frac{{{\rm{\Phi }}}_{0}}{{\rm{A}}},$$

with 1*σ* uncertainties

7 $$\delta M=(\begin{array}{ccc}17.8 & 0.9 & 2.8\\ 0.6 & 8.9 & 0.8\\ 0.8 & 15.1 & 0.4\end{array})\,{\rm{ \% }}.$$

With the inductance matrix we can convert the desired magnetic fluxes threading the left (Φ_l_), middle (Φ_m_), and right (Φ_r_) SQUIDs to the currents applied to the left (*I*_l_), middle (*I*_m_), and right (*I*_r_) flux bias lines as

8 $$(\begin{array}{c}{I}_{{\rm{l}}}\\ {I}_{{\rm{m}}}\\ {I}_{{\rm{r}}}\end{array})={M}^{-1}\,(\begin{array}{c}{{\rm{\Phi }}}_{{\rm{l}}}\\ {{\rm{\Phi }}}_{{\rm{m}}}\\ {{\rm{\Phi }}}_{{\rm{r}}}\end{array}).$$

Here, the positions refer to the positions of the SQUIDs and the flux bias lines in Fig. 1(b).

### Data availability

The datasets generated during and/or analysed during the current study are available from the corresponding author on reasonable request.

## Electronic supplementary material

Supplementary Information
