# Supplementary material for: Flux-tunable phase shifter for microwaves
Source: Sci Rep. 2017 Nov 7;7:14713. doi: 10.1038/s41598-017-15190-2 (PMC5676951; doi:10.1038/s41598-017-15190-2)
Supplement: Supplementary file 1 — Supplementary Information [file 41598_2017_15190_MOESM1_ESM.pdf]

Flux-tunable phase shifter for microwaves

Roope Kokkoniemi, Tuomas Ollikainen, Russell E. Lake, Sakari Saarenpää, Kuan Y. Tan, Janne I. Kokkala, Ceren B. Dağ, Joonas Govenius, and Mikko Möttönen

FORWARD TRANSMISSION COEFFICIENT FOR THE VOLTAGE

In this section, we derive the transmission coefficient of the three-SQUID linear phase shifter considered in the main text. Although identical results can be obtained using classical circuit theory, we choose to work within quantum mechanics [1].

Let us first consider a single LC oscillator interrupting an infinite transmission line at position $x = 0$ as shown in Fig. S1. We define charge operators $\hat{Q}_{\rm a}(x,t)$ for $x \leq 0$ and $\hat{Q}_{\rm b}(x,t)$ for $x \geq 0$ corresponding to the total charge on the transmission line to the left of point x at time t. The corresponding current and voltage operators are given by $\hat{I}_{\rm a/b}(x,t) = -\partial_t \hat{Q}_{\rm a/b}(x,t)$, and $\hat{V}_{\rm a/b}(x,t) = -\frac{1}{c}\partial_x \hat{Q}_{\rm a/b}(x,t)$, respectively, where c is the capacitance of the transmission line per unit length. The Lagrangian density of the system can be expressed as

$$\hat{\mathcal{L}} = \theta(-x)\left\{\frac{l}{2}\left[\partial_t \hat{Q}_a(x,t)\right]^2 - \frac{1}{2c}\left[\partial_x \hat{Q}_a(x,t)\right]^2\right\} + \delta(x)\left(\frac{1}{2}L\hat{I}_{\rm L}^2 - \frac{1}{2}C\hat{V}^2\right) + \theta(x)\left\{\frac{l}{2}\left[\partial_t \hat{Q}_b(x,t)\right]^2 - \frac{1}{2c}\left[\partial_x \hat{Q}_b(x,t)\right]^2\right\}, \quad \text{(S.1)}$$

where l is the inductance per unit length of the transmission line, $\hat{I}_{\rm L}$ is the operator for the current flowing through the inductor, and $\hat{V}$ is the operator for the voltage across the oscillator. The voltage operator $\hat{V}$ can be written in terms of the charge operators as

$$\hat{V} = -\frac{1}{c}\partial_x\left(\hat{Q}_a - \hat{Q}_b\right), \quad \text{(S.2)}$$

and similarly the current through the inductor can be expressed as

$$\hat{I}_{\rm L} = \partial_t \hat{Q}_a + \frac{C}{c}\partial_t\partial_x\left(\hat{Q}_a - \hat{Q}_b\right). \quad \text{(S.3)}$$

Here, we have employed the current–voltage relation for the capacitor C.

Applying the Euler–Lagrange equation for the Lagrangian density in equation (S.1) yields

$$\frac{1}{c}\partial_x\left(\hat{Q}_a - \hat{Q}_b\right) = -L\left[\partial_t^2 \hat{Q}_a + \frac{C}{c}\partial_t^2\partial_x\left(\hat{Q}_a - \hat{Q}_b\right)\right], \quad \text{(S.4)}$$

for $x = 0$. In addition, we find that the charge operators $\hat{Q}_{\rm a}$ and $\hat{Q}_{\rm b}$ satisfy the wave equation for $x \neq 0$.

The Fourier expansions of the charge operators are given by

$$\hat{Q}_{\rm a}(x,t) = \left(\frac{\hbar}{4\pi Z_0}\right)^{\frac{1}{2}} \int_{-\infty}^{\infty} {\rm d}\omega \frac{1}{\sqrt{|\omega|}}\left[\hat{a}_{\rm L}(\omega)e^{-i\omega(t+\frac{x}{v})} + \hat{a}_{\rm R}(\omega)e^{-i\omega(t-\frac{x}{v})}\right], \quad \text{(S.5)}$$

and

$$\hat{Q}_{\rm b}(x,t) = \left(\frac{\hbar}{4\pi Z_0}\right)^{\frac{1}{2}} \int_{-\infty}^{\infty} {\rm d}\omega \frac{1}{\sqrt{|\omega|}}\left[\hat{b}_{\rm L}(\omega)e^{-i\omega(t+\frac{x}{v})} + \hat{b}_{\rm R}(\omega)e^{-i\omega(t-\frac{x}{v})}\right], \quad \text{(S.6)}$$

where $Z_0 = \sqrt{\frac{l}{c}}$ is the characteristic impedance of the transmission line, $v = \sqrt{\frac{1}{lc}}$ is the velocity of the wave in the transmission line, and $\hbar$ is the reduced Planck constant.

Insertion of the above expansions of the charge operators into equation (S.4) yields for any $\omega > 0$

$$Z_0\left[\hat{a}_{\rm L} - \hat{a}_{\rm R} - \left(\hat{b}_{\rm L} - \hat{b}_{\rm R}\right)\right] = i\omega L\Big\{\left(\hat{a}_{\rm L} + \hat{a}_{\rm R}\right) - i\omega C Z_0\left[\hat{a}_{\rm L} - \hat{a}_{\rm R} - \left(\hat{b}_{\rm L} - \hat{b}_{\rm R}\right)\right]\Big\}. \quad \text{(S.7)}$$

The electric charge must be conserved, $\partial_t \hat{Q}_{\rm a}(x=0,t) = \partial_t \hat{Q}_{\rm b}(x=0,t)$, which can be written in terms of the annihilation operators as

$$\hat{a}_{\rm L} + \hat{a}_{\rm R} = \hat{b}_{\rm L} + \hat{b}_{\rm R}. \quad \text{(S.8)}$$

The SQUID-based phase shifter consists of three systems similar to the one we analyzed above, see Fig. S2.

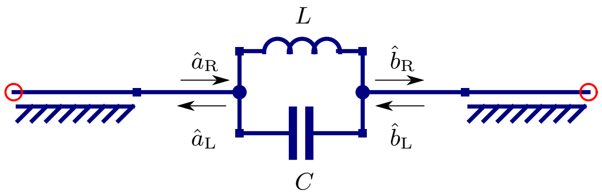

FIG. S1. Transmission line interrupted by an LC oscillator. The operators $\hat{a}_{\rm L/R}$ and $\hat{b}_{\rm L/R}$ correspond to annihilation an excitation of the mode propagating to the left (L) or to the right (R) for $x < 0$ ($\hat{a}$) and $x > 0$ ($\hat{b}$).

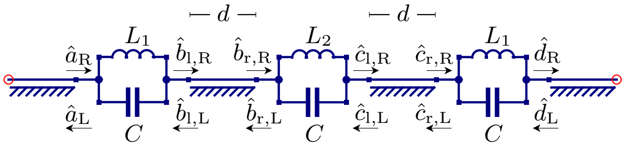

FIG. S2. Three LC oscillators interrupting a transmission line. Inductors on the left and right have an equal inductance L_1, and the inductor in the middle has an inductance L_2. All capacitors have an equal capacitance C. The inductors are separated by the distance d. The operators $\hat{a}_{\mathrm{L/R}}$, $\hat{b}_{\mathrm{l/r,L/R}}$, $\hat{c}_{\mathrm{l/r,L/R}}$, and $\hat{d}_{\mathrm{L/R}}$ are annihilation operators for excitations of the mode propagating leftwards (L) or rightwards (R) at the left (l) or right (r) end of a transmission line connecting two LC oscillators.

Thus, the boundary conditions are similar to equations (S.7) and (S.8) and they are given by

$$Z_0\left(\hat{a}_{\mathrm{L}}-\hat{a}_{\mathrm{R}}-\hat{b}_{\mathrm{l,L}}+\hat{b}_{\mathrm{l,R}}\right)=i\omega L_1\Big[\hat{a}_{\mathrm{L}}+\hat{a}_{\mathrm{R}} -i\omega CZ_0\Big(\hat{a}_{\mathrm{L}}-\hat{a}_{\mathrm{R}}-\hat{b}_{\mathrm{l,L}}+\hat{b}_{\mathrm{l,R}}\Big)\Big], \tag{S.9}$$

$$Z_0\left(\hat{b}_{\mathrm{r,L}}-\hat{b}_{\mathrm{r,R}}-\hat{c}_{\mathrm{l,L}}+\hat{c}_{\mathrm{l,R}}\right)=i\omega L_2\left[\hat{b}_{\mathrm{r,L}}+\hat{b}_{\mathrm{r,R}} -i\omega CZ_0\left(\hat{b}_{\mathrm{r,L}}-\hat{b}_{\mathrm{r,R}}-\hat{c}_{\mathrm{l,L}}+\hat{c}_{\mathrm{l,R}}\right)\right], \tag{S.10}$$

$$Z_0\left(\hat{c}_{\mathrm{r,L}}-\hat{c}_{\mathrm{r,R}}-\hat{d}_{\mathrm{L}}+\hat{d}_{\mathrm{R}}\right)=i\omega L_1\Big[\hat{c}_{\mathrm{r,L}}+\hat{c}_{\mathrm{r,R}} -i\omega CZ_0\Big(\hat{c}_{\mathrm{r,L}}-\hat{c}_{\mathrm{r,R}}-\hat{d}_{\mathrm{L}}+\hat{d}_{\mathrm{R}}\Big)\Big], \tag{S.11}$$

$$\hat{a}_{\mathrm{L}}+\hat{a}_{\mathrm{R}}=\hat{b}_{\mathrm{l,L}}+\hat{b}_{\mathrm{l,R}}, \tag{S.12}$$

$$\hat{b}_{\mathrm{r,L}}+\hat{b}_{\mathrm{r,R}}=\hat{c}_{\mathrm{l,L}}+\hat{c}_{\mathrm{l,R}}, \tag{S.13}$$

and

$$\hat{c}_{\mathrm{r,L}}+\hat{c}_{\mathrm{r,R}}=\hat{d}_{\mathrm{L}}+\hat{d}_{\mathrm{R}}. \tag{S.14}$$

In addition to the above boundary conditions, the operators annihilating the same excitation of a mode, but at different locations of the same transmission line, are related by a phase shift. We obtain the corresponding relations from the Fourier expansions of the charge operators as

$$\hat{b}_{\mathrm{r,L}}=\hat{b}_{\mathrm{l,L}}e^{-i\varphi}, \tag{S.15}$$

$$\hat{b}_{\mathrm{r,R}}=\hat{b}_{\mathrm{l,R}}e^{i\varphi}, \tag{S.16}$$

$$\hat{c}_{\mathrm{r,L}}=\hat{c}_{\mathrm{l,L}}e^{-i\varphi}, \tag{S.17}$$

$$\hat{c}_{\mathrm{r,R}}=\hat{c}_{\mathrm{l,R}}e^{i\varphi}, \tag{S.18}$$

where $\varphi=\omega\frac{d}{v}$ is the phase shift owing to a transmission line connecting two LC oscillators.

From the linear system of equations (S.9)–(S.18), we can solve $\hat{d}_{\mathrm{R}}$ in terms of $\hat{a}_{\mathrm{R}}$ and $\hat{d}_{\mathrm{L}}$, i.e., how the output is affected by the inputs. This yields

$$\hat{d}_{\mathrm{R}}=\frac{1}{g}\left[\hat{a}_{\mathrm{R}}f+\hat{d}_{\mathrm{L}}f_{\mathrm{R}}\right], \tag{S.19}$$

where f and g are given by

$$f(\omega)=8Z_0^3e^{2i\varphi}\left(CL_2\omega^2-1\right)\left(CL_1\omega^2-1\right)^2, \tag{S.20}$$

and

$$g(\omega)=\big\{4Z_0^2\big(CL_2\omega^2-1\big)\big(CL_1\omega^2-1\big)+L_2L_1\omega^2\times\big(e^{2i\varphi}-1\big)+2i\omega Z_0\big[CL_2L_1\omega^2\big(2+e^{2i\varphi}\big)-L_2 -L_1\big(1+e^{2i\varphi}\big)\big]\big\}\big[2Z_0\big(CL_1\omega^2-1\big) -iL_1\omega\big(e^{2i\varphi}-1\big)\big]. \tag{S.21}$$

The factor f_{R} assumes the form

$$f_{\mathrm{R}}=\omega\Big\{-2iL_2e^{2i\varphi}\left[2Z^2\left(CL_1\omega^2-1\right)^2+L_1^2\omega^2\right] +L_1\left[L_2\omega-2iZ_0\left(CL_2\omega^2-1\right)\right]\left[2Z_0\left(CL_1\omega^2-1\right) +iL_1\omega\right]-iL_1e^{4i\varphi}\left[2Z_0\left(CL_2\omega^2-1\right)-iL_2\omega\right] \times\left[2Z_0\left(CL_1\omega^2-1\right)-iL_1\omega\right]\Big\}. \tag{S.22}$$

From the equations (S.19)–(S.21) we obtain the scattering parameter S_{21}, or the transmission coefficient

$$S_{21}\equiv\left(\frac{\partial\hat{d}_{\mathrm{R}}}{\partial\hat{a}_{\mathrm{R}}}\right)^*=\frac{f^*}{g^*}, \tag{S.23}$$

which coincides with equation (2) in the main text. Since the circuit is linear, by symbolically calculating the partial derivative, we obtain the complex coefficient which transforms photons in mode $\hat{a}_{\mathrm{R}}$ to those in mode $\hat{d}_{\mathrm{R}}$. This enables direct comparison between the theory and the experiments since we can measure the transmission coefficient.

In equation (S.23), we define the transmission coefficient as a complex conjugate of the derivative, since this was observed to match with the measured transmission coefficients. We attribute the conjugation to the freedom of choice of the sign of the exponent in the Fourier transform. Manufacturer of our measurement equipment has likely chosen different sign than we have. Equivalently, if heterodyne detection is used, the sign of the phase changes depending on whether the frequency of the local oscillator is chosen to be higher or lower than the frequency of the measured signal.

Let us derive equations (3) and (4) of the main text. For the phase shifter to exhibit full transmission, the reflection coefficient must vanish, i.e., $f_{\mathrm{R}}=0$. Thus equation (S.22) yields

$$L_2=\frac{2L_1Z_0}{X}\left[2Z_0\cos(2\varphi)\left(CL_1\omega^2-1\right)+L_1\omega\sin(2\varphi)\right], \tag{S.24}$$

where

$$\begin{aligned} X =& L_1\omega^2\cos(2\varphi)\left[L_1\left(4C^2\omega^2 Z_0^2-1\right)-4CZ_0^2\right] \\ & -4CL_1\omega^2 Z_0^2+2L_1\omega Z_0\sin(2\varphi)\left(2CL_1\omega^2-1\right) \\ & +L_1^2\omega^2+2Z_0^2+2C^2L_1^2\omega^4 Z_0^2. \end{aligned} \tag{S.25}$$

Since we are looking for parametrizations for the inductances which yield full transmission, we may write

$$S_{21}=\frac{f^*}{g^*}=e^{i(\theta-2\varphi)}. \tag{S.26}$$

By substituting equations (S.20), (S.21), (S.24), and (S.25) into equation (S.26) we obtain equation (3) of the main text. Furthermore, an insertion of equation (3) of the main text into equation (S.24) yields equation (4) of the main text.

[1] B. Yurke and J. S. Denker, Phys. Rev. A **29**, 1419 (1984).
